# Transition support arrangements to support new graduate & novice nurses entry into perioperative nursing: A scoping review

**DOI:** 10.1016/j.heliyon.2023.e23316

**Published:** 2023-12-13

**Authors:** Nick Nijkamp, Pauline Calleja, Ashlyn Sahay

**Affiliations:** CQUniversity: School of Nursing, Midwifery and Social Sciences, Queensland, Australia

**Keywords:** Education, Graduate nurses, Novice nurse, Orientation, Perioperative nursing, Scoping review, Transition programs, Transition to practice

## Abstract

**Background and objectives:**

As novice nurses transition into the workforce, they often experience transition shock as they assimilate into a new role, causing cause significant stress, anxiety and job dissatisfaction. This phenomenon is commonly observed within the perioperative nursing speciality. The development of transition support programs is aimed at assisting novice nurses' transition by providing fundamental knowledge, socialisation, support, and training.

This review aimed to uncover the support programs and their components available to nurses transitioning into the perioperative speciality. The research question that provided guidance for this review was *‘What are the transition* support *arrangements, and their characteristics, to support new graduate nurses and novice nurses who are transitioning into perioperative nursing?'.*

**Literature search:**

Arksey & O'Malley's’ five-step scoping review framework was used. The researchers performed a comprehensive literature search of PubMed, Proquest, CINHAL and SCOPUS with no limit on publication date until April 2023. A blinded screening process was undertaken, and the data extraction was tabulated. Data was presented as a narrative synthesis following thematic analysis.

**Results:**

The initial search identified 537 publications. Screening and duplicate removal led to the exclusion of 512 publications. Of the 25 publications included in this review, two were primary research publications, while the other 23 were discussion papers. Analysis indicated that program approaches and components of programs were frequently described.

**Conclusion:**

The findings highlight the significance of transition programs within the perioperative speciality area. However, the paucity of empirical evidence on the pedagogical underpinnings and evaluation of effectiveness indicates the need for further research. Conducting further research within perioperative transition to practice will enable programs to be designed based on theoretically-sound and evidence-based approaches to support nursing transition to practice within the speciality perioperative environment.

## 1 Background

### 1.1 Challenges during transition into nursing

Newly graduated nurses or those moving to a different specialty undergo a significant transformation and transition phase. These nurses are often referred to as new graduate registered nurses or novice registered nurses (hereafter referred to as novices) as they are new to a work environment or practice setting. Many novice nurses experience *transition shock,* which describes the emotional and physical stressors experienced during assimilation into a new environment [1]. During transition, novices describe their experiences as “*drowning*”, “*terrified*” or “*scared to death*” [1]. During the transition phase, nurses are also likely to experience fatigue, exhaustion, and burnout. This phenomenon is well-established in international seminal literature [2,3]. Contemporary literature estimates that 35 %–60 % of new graduate nurses leave the first position within one year of commencing in the role [4]. This is predominantly attributed to the effects of transition shock and the theory-practice gap, which is especially prevalent in specialised areas [1,4].

### 1.2 Transitioning into the perioperative nursing speciality

To thrive within the perioperative environment, a high level of procedural knowledge, specialised equipment knowledge, psychomotor skills, and interpersonal skills are required. Professional bodies such as the Australian College of Perioperative Nursing (ACORN) [5] and the Association of PeriOperative Registered Nurses (AORN) [6] recognise the unique, complex and often challenging environment of the perioperative speciality. These professional organisations recommend the formal support of novice perioperative nurses to build the specialised knowledge, clinical competence and attitudes to be a safe, effective, competent and confident perioperative nurses [5]. Additionally, it is recommended that these skills and knowledge are acquired through planned education programs, incorporating clinical practice and theoretical learning, provided by experienced educators, mentors and preceptors specialising in perioperative nursing [5]. This review defines perioperative nurses as nurses who care for patients in the immediate pre-operative, intra-operative and post-operative settings.

### 1.3 Role of transition programs

The accepted view in the literature is that transition to practice programs are necessary (also referred to as: orientation programs, transition arrangements, residencies or internships) [4]. The primary objective of transition programs is to support novice nurses to bridge the knowledge gap during their transition phase [7]. Secondary objectives of these programs include socialisation within the professional environment, assimilation into the cultural milieu, development of confidence and competence and lessening the effects of transition shock [4,7]. Transition programs are developed to equip novice nurses with the knowledge, skills and confidence to be competent and proficient clinicians. This is achieved through the use of various teaching methods and pedagogies, such as; didactic learning, simulation, reflection and assessments [8].

### 1.4 Challenges in transition programs and purpose of review

Transition programs for novice nurses play a pivotal role in facilitating their integration into healthcare settings and the development of competent and confident nurses. However, these programs face challenges. A review of transition programs offered through healthcare providers uncovered that programs are unmonitored and vary significantly in the content provided [9]. There is no standardisation regarding their duration, level of supervision provided to novice nurses, specific theoretical knowledge taught, or the qualifications held by the educator/preceptor [9]. This makes it difficult to ascertain which programs and program components effectively support novice nurses' transition, despite substantial literature published on transition arrangements.

In response to these challenges, this scoping review was conducted to identify if there are gaps in the literature related to perioperative nursing transition arrangements. By mapping existing literature, this review aimed to determine what program components and approaches are available to support novice nurses transitioning into the perioperative nursing speciality.

## 2 Method

This scoping review aimed to examine and map empirical research and discussion publications to identify knowledge gaps and map the known support mechanisms for novice nurses' transition into perioperative nursing practice. The review followed a five-step scoping review framework which included identifying the research question, identifying relevant studies, study selection, data extraction and synthesis, and collating, summarising, and reporting results [10]. This framework was chosen as it supports the collection, synthesis, and reporting of a variety of resources, including discussion papers which are important due to the limited empirical evidence available. The discussion papers included within this review add valuable information to this topic area.

**Fig. 1:**
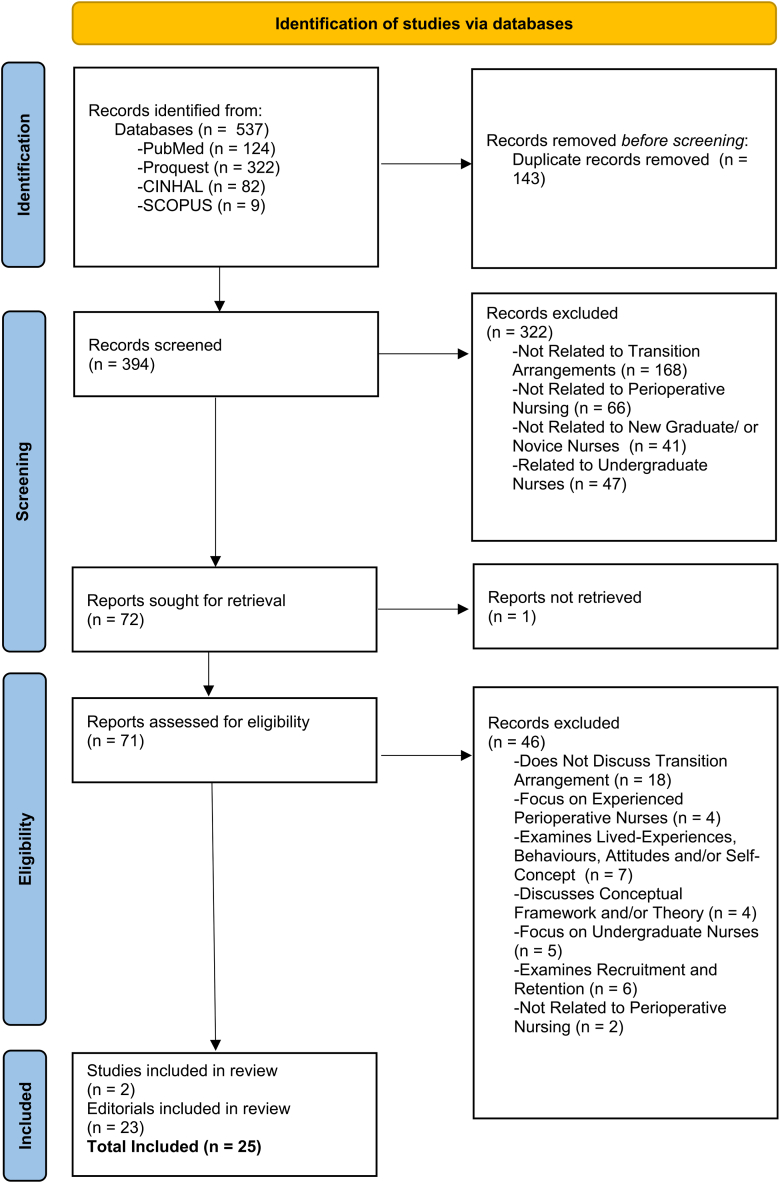
Preferred reporting items for systematic reviews and meta-analyses (PRISMA) four-phase flow diagram.

The research question (RQ) guiding this review was *‘What are the transition* support *arrangements, and their characteristics, to support new graduate nurses and novice nurses who are transitioning into perioperative nursing?*’. A systematic approach was used to search four databases; ProQuest, PubMed, Cumulative Index of Nursing and Allied Health Literature (CINAHL), and SCOPUS. Fig. 1 depicts the Preferred Reporting Items for Systematic Reviews and Meta-Analyses (PRISMA) Four-Phase Flow Diagram [11], demonstrating the systematic process used for this scoping literature review. Additionally, the Preferred Reporting Items for Systematic Reviews and Meta-Analyses extension for Scoping Reviews (PRISMA-ScR) checklist was followed [12].

### 2.1 Inclusion/Exclusion criteria

Pertinent inclusion and exclusion criteria were used to ensure that the scoping review captured a comprehensive range of literature. Table 1 demonstrates the inclusion and exclusion criteria used to guide this literature search. Editorial and discussion publications were included in the review as the preliminary searches indicated a need for more empirical research on this topic. Additionally, no age limit was set on the publications included within this review as the inclusion of all papers holds potential for future analysis of trends within perioperative transition programs. The search also considered international literature, as the transition to nursing practice is well-documented worldwide, provided the articles were written in English.

**Table 1:** Inclusion and Exclusion criteria used to guide scoping literature review.

Inclusion Criteria	Exclusion Criteria
• English, or officially translated. • Peer-reviewed. • Full text only. • Papers were searched until April 2023, with no age limit set on previous publications. • Literature reviews, discussions, editorials, and primary research (any methodology). • Studies that incorporate post-graduate degrees (certificates and diplomas) are also included. • Literature that discusses nurses who enter the perioperative speciality from other backgrounds, overseas or who have completed re-entry programs, as these nurses may potentially require a transition arrangement to support their assimilation.	• Papers not related to the perioperative nursing speciality. • Papers related to undergraduate training in perioperative nursing. • Papers that discussed experienced perioperative nurse training. • Papers not related to transition support arrangements (this includes education programs that are ongoing professional development rather than transition support).

### 2.2 Search strategies

The PICo (Population, Interest/Intervention, Context) framework was used to develop the research question and relevant keywords and synonyms were included to comprehensively search the databases (refer to Table 2). Search terms were selected based on previous literature and MeSH Headings. Search strings included the Boolean operators of ‘OR’ and ‘AND’. Search strings used in database searches included; *"(Novice nurse OR new graduate nurse) AND perioperative AND transition”*, "(*Novice nurse OR new graduate nurse) AND perioperative AND orientation*”, "(*Novice nurse OR new graduate nurse) AND perioperative AND support*”, *"(novice nurse OR graduate nurse) AND perioperative AND (transition OR orientation OR support)*", and, "(New graduate nurs* OR novice nurs*) AND perioperative AND support".

**Table 2:** PICo – summarisation of search terms.

		Keyword	Synonyms
**P**	Population	(New Graduate Nurses) OR (Novice Nurses)	New graduates, new graduate registered nurses, neophyte nurse.
**I**	Interest/Intervention	Transition to Practice	Transition to practice program, transition to practice arrangement, graduate support program, internship, new graduate support program, nurse residency, mentorship, preceptorship, preceptorship program, orientation, transition.
**Co**	Context	Perioperative	Surgical, operating theatre, operating room, theatre, operating suite, anaesthetics, perianaesthesia.

Search results were extracted and exported into EndNote. These were then exported into Rayyan [13] for blinded screening. Blinded screening was completed by two researchers, with a third researcher moderating and resolving any conflicts that occurred. Full-text screening was completed by the lead researcher, with spot-checking and moderation completed by other authors.

### 2.3 Data extraction and synthesis

Braun and Clarke's [14] Six-Step Guide to Thematic Analysis was used to extract and analyse the data from the retrieved publications. Data extraction included the collection of publication information (author/s, publication year, country), aims and objectives of the publication, the key components and approaches of programs described within the publication, assessments embedded within programs, transition program length, and limitations of the publication. Data analysis and mapping involved identifying common elements and themes within the data and developing overarching themes, in order to address the research question. The lead researcher conducted the analysis, and a structured reporting table was used to synthesise the findings. Table 3 contains the structured reporting table and demonstrates the common themes found across transition programs.

**Table 3:** Summarisation of transition components and approaches within the literature.

Citation		Country	Transition Length	Transition Arrangements		Transition Components			Additional Information
				Didactic Components	Professional Organisation Courses	Simulation	Preceptorship/Mentorship	Assessment	
**Peer-Reviewed Discussions**									
(Byrd et al., 2015)	US		1 Week Orientation and 4–6 Month Transition	✓	✓ AORN Periop 101 Course	✓	✓	✓	-Organisational familiarisation (hospital-wide orientation)
(Ceschini, 2016)	US		8 Week Classroom, 7 Month Orientation	✓ Lectures, discussions and videos		✓ Hands-on-training	✓	✓ Weekly quiz and clinical skills assessment	
(Dajee, 2002)	UK		Not Discussed	✓		✓			-Group work -Organisational familiarisation (guided tours) -Reflective practice and monthly debrief
(DeKastle, 2010)	US		6–9 Months	✓	✓ AORN Periop 101 Course	✓	✓	✓	-Workplace cultural integration -Reflective practice (debriefing) -Organisational familiarisation (scavenger hunt) -Observation.
(Fitzgerald, 2009)	US		12 Weeks	✓		✓	✓	✓	-Program based on Benner's Novice to Expert Theory and Adult Learning Principles
(Graling & Rusynko, 2001)	US		6 Months	✓ 160 h of classroom time			✓	✓ Biweekly Quiz	-Reflective practice (learning journal) -Computer Based Training
(Graling & Rusynko, 2004)	US		Not Discussed	✓ 160 h classroom time		✓			-Variety of teaching techniques used -Reflective practice (journaling)
(Gorgone et al., 2016)	US		7 Week Orientation and 9–12 Month Transition	✓	✓ AORN Periop 101 Course	✓	✓	✓	-Procedure Log -Reflective practice (journaling and goal setting) -Experiential Learning Opportunities -Organisational familiarisation (scavenger hunt)
(Koorey, 2016)	NZ		Not Discussed	✓ Online learning program			✓	✓ Formative assessments	-Role play to teach clinical skills
(Latz & Nordbye, 2004)	US		Either: 10 Weeks or 2 Weekends (Condensed)	✓		✓ Hands-on laboratory		✓ Formative assessments and oral quiz	
(Martin, 2011)	US		30 Weeks	✓ 5 weeks of didactic learning	✓ AORN Periop 101 Course		✓	✓ Online Test and logbook	−25 weeks with clinical partner
(Matapo & Kennedy, 2020)	NZ		12 Weeks	✓		✓	✓		-Company representative involvement -Discussions
(Osgood & Hemingway, 2019)	US		20 Week		✓ AORN Periop 101 Course	✓	✓	✓	
(Penprase, 2000)	US		11 Weeks	✓ Group discussions and presentations			✓	✓	
(Persaud, 2008)	US		7–9 Months	✓			✓		
(Punnara & Barta, 2009)	US		12 Weeks	✓			✓	✓ Weekly evaluations and clinical competencies	
(Ray et al., 2015)	US		9 Months	✓					-Organisational familiarisation (visiting other units)
(Saver, 2014a)	US		12 Weeks	✓			✓		
(Saver, 2014b)	US		1 Year	✓	✓ AORN Periop 101 Course	✓ Mock operating room		✓ Regular evaluations	-Shadowing team members
(Whelan et al., 2016b)	CA		Not Discussed	✓ Classroom learning and lectures		✓			
(Wilson, 2012)	US		12 Weeks		✓ AORN Periop 101 Course		✓		-Social support -Reflective practice (debrief and experience sharing)
(Wood, 2015)	US		6 Months	✓ Six module program			✓		
(Wu & Taylor, 2020)	NZ		12 Weeks	✓		✓ ‘Simulate & Pause’ scenarios	✓	✓ Competency assessments	
**Research Papers**									
(Richardson-Tench & Martens, 2005) Exploratory, descriptive study.	AU		Not Discussed	✓				✓ Written examination, case study presentation and clinical performance tool	
(Kuiper, 2004) Comparative descriptive design and narrative analysis.	US		9 Weeks	✓			✓		-Reflective practice (journaling)

## 3 Results

The literature search yielded 537 publications, 143 were removed as duplicates. First stage screening (title and abstract) led to the removal of 322 publications. One publication was excluded due to the unavailability of the full-text. Full-text screening led to the removal of 46 publications. Twenty-five publications (2 research publications and 23 discussions) were included in this review and were from five countries (refer to Table 3). Most (n = 19) publications came from the United States of America (USA), with other publications from Australia, the United Kingdom, Canada, and New Zealand.

The two empirical research publications were qualitative. The first was an exploratory, descriptive study that used a reflective method to evaluate the teaching approach within a perioperative specialised Graduate Diploma [15]. The second research publication used a comparative descriptive design and narrative analysis to examine self-regulated learning strategies [16]. The remaining publications were discussions (n = 23), of which the majority described transition programs offered by various institutions. Additionally, 64 % (n = 16) of the discussion papers described participant demographics that have completed or were currently completing the program. Table 3 presents an overview of the various educational approaches used within transition programs. The various findings within transition arrangements were classified into two themes: 1. Transition Program Approaches and 2. Transition Program Components. Overall, the scoping review provides insight into the current state of transition programs for novice nurses in the perioperative setting and highlights areas for future research and improvement in the design and implementation of these programs.

Theme 1 – Transition Program Approaches

A prominent theme that emerged from the literature related to approaches used within transition programs. Within this review, an approach is defined as an overarching strategy that guides the development and implementation of the program. Approaches outline and influence the overall educational methods and components embedded within programs. The review uncovered five approaches: didactic education, professional organisation courses, organisation familiarisation, reflective practice and workplace culture integration.

### 3.1 Didactic education

Didactic education was incorporated the majority (n = 23) of the publications discussing perioperative transition programs, aimed to aid novice nurses' assimilation and knowledge development (refer to Table 3). Didactic education encompasses several specific teaching methods, including lectures, discussion groups, videos, classroom learning, presentations, and in-services. One program also used didactic education provided by medical company representatives to deliver content specific to surgical equipment [17].

One publication discussed the underpinning theoretical and conceptual frameworks used within their transition program [18]. This program addresses the differing generational, educational and life experiences of the novice perioperative nurses completing the program and details the incorporation of Benners' Novice to Expert Theory [18]. While didactic education approaches are popular among transition programs, there was an overarching lack of rationale or explanation of why they had been chosen, and a paucity of evaluation regarding its effectiveness as a pedagogical teaching strategy.

### 3.2 Professional organisation courses

The literature search yielded seven publications incorporating professional organisation courses into their transition programs. Seven publications based in the USA highlighted the use of the Association of Perioperative Registered Nurses (AORN) *Periop 101: A Core Curriculum*^*TM*^ *Course* (*AORN Periop 101*) within their relevant support program. This e-learning course incorporates theoretical learning and clinical experiences [19]. Six discussions included clinical experiences and preceptorship in conjunction with the *AORN Periop 101* program [[19], [20], [21], [22], [23], [24]]. Additionally, one facility has aligned and tailored the AORN program by changing the course content from adult-specific to paediatric-specific [21]. It is claimed that standardised programs, such as the *AORN Periop 101 Course*, have reduced training time for novice nurses, increased quality improvement and reduced attrition rates [19].

### 3.3 Organisational familiarisation

Organisational familiarisation aims to familiarise novice nurses with the perioperative department, and is described in four papers [20,21,25,26]. Organisational familiarisation was aided by guided tours through ancillary departments and other health departments [25,26]. Similarly, scavenger hunts are documented in two publications [20,21] to facilitate organisation familiarisation by seeking and locating equipment and resources.

### 3.4 Reflective practice

Reflective practices such as journaling and logbooks were used within several programs described in the literature (n = 3) [16,21,22,27]. A comparative descriptive study (p = 26) that used narrative analysis methods found that reflective journaling has the potential to improve critical thinking, enhance decision making and support competency development [16]. In addition, two programs required novice nurses to keep a logbook of procedures they have performed [21,22]. Progress evaluation and reflection was facilitated using logbooks.

### 3.5 Workplace culture integration

One publication described workplace culture integration as an approach used within transition arrangements. The program described by DeKastle [20] discusses the importance of integration into the workplace cultural milieu as an important contributor of novice nurse success within the perioperative setting. This program used various methods to achieve cultural integration, including ice breaker exercises, group introductions and a meet and greet with department leaders.

Theme 2 – Transition Program Components

Another emerging theme that was uncovered pertains to program components. These components are the fundamental building blocks contributing to an educational program's efficacy and comprehensive nature. Within this review, components relate to specific education strategies that have been described in the literature. Commonly, an educational approach may have multiple components. Transition program components that were identified within this review include program length, preceptorship and mentorship and simulation.

### 3.6 Program length

Program length is an objective measure of time spent within the transition space and an important component of the program itself. Twenty papers discussed the length of their transition programs, as shown in Table 3. Of the transition programs compared, the lengthiest ran for twelve months (Table 3) [21,28]. The shortest program extended over ten weeks, including two evening classes per week, or condensed into two weekends [29].

Further analysis of program length indicated that some (n = 3) incorporated a ‘classroom’ time or orientation prior to clinical experience and immersion within the perioperative environment (Table 3) [19,21,30]. Classroom time and orientation were used to introduce the organisation and perioperative nursing theory.

### 3.7 Preceptorship and mentorship

Numerous publications (n = 18, see Table 3) discussed using preceptors or mentors within their transition program to support and educate novice nurses in clinical practice [11,22,31]. One publication described using the Learning Style Inventory to match novice nurses with a compatible preceptor based on learning and teaching style [21].

Two publications within the literature also described the training requirements of preceptors [21,31]. These programs offer a training program for experienced Registered Nurses to equip them with the knowledge and skills to precept novice nurses within the perioperative nursing speciality.

### 3.8 Simulation

Simulation and hands-on teaching are pedagogical strategies that allows the transference of theoretical knowledge applied to real-world scenarios in a safe, controlled and supported environment [23]. Thirteen publications within the review describe the incorporation of simulation or hands-on teaching (refer to Table 3). Some programs (n = 4) incorporated a skills laboratory or mock operating theatre experience into their program to practice skills [20,29,30,32]. Three programs were simulation-intensive [23,27,33]. The remaining publications (n = 6) briefly described the inclusion of simulation pedagogies within their transition programs. Simulation has been reported as a method of increasing novice nurses' confidence when dealing with common perioperative emergencies [19].

### 3.9 Assessment within programs

Whether formative or summative, assessment within transition programs acts as an integral component within transition support programs. These evaluative milestones allow the participants and others to gauge their overall confidence, competence and knowledge throughout their transition into perioperative nursing. Fifteen publications (60 %) discuss assessments embedded within transition programs.

A multitude of assessment strategies have been identified within the publications. The publications indicate that the most common assessment methods included; examinations [19], quizzes [27,31], clinical skills and clinical competence assessments [20,23,30,31,34,35], preceptor evaluation [23,28,31,32], oral test [29] and simulation assessment [28,29].

## 4 Discussion

The review aimed to establish the scope of transition programs that target novice perioperative nurses entering the speciality. The results reveal a broad range of transition programs, with various components and approaches embedded to facilitate assimilation and the development of knowledge and skills in this specialised area. The available literature emphasises the significance of transition support programs across all nursing specialities in the development of competent and confident nurses [7].

Difficulty transitioning emanates within the perioperative nursing realm, as novice perioperative nurses report anxiety, inadequacy, and insecurity throughout the transition period [1]. A two-year longitudinal study uncovered that novice nurses’ stress and anxiety are highest in the first two months of transitioning to employment and diminish as confidence increases [36]. The perioperative speciality is further complicated by: specialised knowledge requirements, advanced clinical skills, ability to use complex technology, and, high levels of interpersonal and communication skills [4,37,38]. Many novice nurses seek employment in a transition program to address these challenges [4]. The reviewers determined that transition programs have an integral role in lessening the effects of anxiety and transition shock by providing the specialised knowledge and skills needed to be proficient nurses, which is supported in the literature [39].

To ensure the effectiveness of transition programs for novice perioperative nurses, programs should be developed based on sound pedagogical strategies and adult learning principles. Each identified component has a pedagogical underpinning and may present an effective teaching technique to aid novice nurses' assimilation and knowledge development within the perioperative speciality area. For instance, simulation is a known pedagogy used within undergraduate nursing training [40]. Arguably, any of the transition components or approaches identified within this review could be used to develop a transition arrangement. Regarding the evaluation of programs, publications within this review do not discuss the efficacy of the pedagogical components. The papers retrieved were predominantly descriptive rather than evaluative, making it difficult to establish the efficacy of practices within transition programs. The review highlights the need for more discussion surrounding the rationale behind the use of these components.

Twelve publications identified in this review discuss recruitment, retention, and staffing shortages and use retention as a measure of program success. This focus is likely due to the staffing shortages and attrition rates that trouble the perioperative nursing sector [[41], [42], [43]]. Transition programs contribute to retention, as one of the goals of transition programs is to retain novice nurses in the perioperative speciality. Therefore, the efficacy of programs is frequently measured by participant retention post-program completion [4]. There should be an acceptable rate of attrition which transition programs can be benchmarked against, as not every nurse is suited to every role. However, a focus on retention creates difficulty in establishing which transition programs are effective in developing competent and confident perioperative nurses.

Significant variability is shown across the various programs and their providers. Broadly, there is no set model for their length, supervision provided, content covered, assessments embedded within the program or the qualifications of the educators. This review shows considerable variation in both program length and the pedagogical content used within programs (Table 3). This review has shown that transition programs do not follow a standardised framework. Although the majority of publications examined in this review have similar aims and desired outcomes from their transition programs. Additionally, consensus on necessary elements of a standardised transition program would support healthcare organisations to create contextualised programs to suit their varying resource capabilities.

In the ‘Australian Educating the Nurse of the Future’ report, Schwartz [9] argues that the variability of transition programs poses a significant challenge in evaluating the outcomes and effectiveness, making it difficult to establish which practices within transition programs are best practice. The future development of transition programs should incorporate empirical evidence and rigorous educational pedagogy, which would require further research to be undertaken in the space. The development of best-practice guidelines or a standardised framework would also strengthen the development and delivery of transition programs. From both an organisational standpoint and a novice nurses' view, the importance of transition programs has been consistently demonstrated in the literature [44].

Notably, the review uncovered a significant gap in empirical research within the literature, with 8 % (n = 2) of the included articles being empirical literature. The remaining articles (n = 22) were descriptive discussion publications. Additional empirical research is required within this field. Research should focus on program component effectiveness to provide the evidence required to underpin the future development of transition programs. Addressing this gap can also potentiate the development of a transition guidelines and a framework to support new perioperative nurses. Findings from this international scoping review can potentially guide future empirical research into the field of perioperative transition to practice.

## 5 Limitations

This scoping literature review contributes to the contemporary knowledge-base of transition to practice, specifically within the perioperative arena, by describing and discussing the support arrangements that are available to novice nurses who are transitioning into this speciality. However, four limitations were noted by the research team. Firstly, there is a paucity of empirical research within the literature. There was also noted to be a deficit in the reporting of the transition programs pedagogical underpinning. Results within this review were also limited by the methods and limitations of the publications included in this review. Lastly, the literature search excluded publications not written in English. The authors acknowledge that there may be publications related to this topic written in other languages.

## 6 Conclusion

Historical and contemporary literature document the difficulties that novice nurses face when transitioning from undergraduate RNs to new graduate or when transitioning into a new speciality. This period is often filled with stress, anxiety, and a significant learning curve to overcome as one attempts to assimilate into a new professional environment. The complex perioperative environment further exacerbates the impact of transition shock. Although the literature recognises the role of transition support programs, there is a paucity of discussion surrounding this topic area within perioperative nursing arena and program evaluation.

This review has partially answered the research question by describing the transition programs, and the approaches, components and assessments within them. However, a significant gap caused by the paucity of empirical literature has been uncovered. Common approaches that were described in the literature included didactic education, professional organisation courses, organisational familiarisation, reflective practice and cultural integration into the workplace. Common components identified within the publications included program length, preceptorship and mentorship and simulation.

The review was unable to gauge the effectiveness of the reported components and approaches due to the descriptive nature of the publications. There is a need to undertake further research within perioperative transition to practice enabling programs to be designed based on empirical evidence and pedagogical theory to effectively support novice nurses’ transition to practice within the speciality environment.

## Funding information

No funding was received for the preparation and writing of this literature review.

## Funding

No funding was received for the preparation and writing of this literature review.

## Ethics declaration

Review and/or approval by an ethics committee was not needed for this study because this study was a scoping literature review conducted through database searches. As such, this study did not use any participants.

## Data availability statement

All data that has been used within this article has been referenced herein.

## CRediT authorship contribution statement

**Nick Nijkamp:** Writing – original draft, Writing – review & editing. **Pauline Calleja:** Supervision, Writing – review & editing. **Ashlyn Sahay:** Supervision, Writing – review & editing.

## Declaration of competing interest

The authors declare that they have no known competing financial interests or personal relationships that could have appeared to influence the work reported in this paper.

## References

[1] Wakefield E. (2018). Is your graduate nurse suffering from transition shock?. Journal of Perioperative Nursing.

[2] Duchscher J., Windey M. (2018). Stages of transition and transition shock. Journal of Nurses in Professional Development.

[3] Kramer M. (1974). United States: C.V.

[4] Van Camp J., Chappy S. (2017). The Effectiveness of Nurse Residency Programs on Retention: A Systematic Review. Aorn J.

[5] (2020). Australian College of Perioperative Nursing, *Standards for Perioperative Nursing in Australia*.

[6] Association of Perioperative Registered Nurses (2022).

[7] Chang E., Daly J. (2020).

[8] Allanson A., Fullbrook P. (2010). Preparation of nurses for novice entry into perioperative practice: evaluation of a short education program. Journal of Perioperative Nursing.

[9] Schwartz S. (2019).

[10] Arksey H., O'Malley L. (2005). Scoping studies: towards a methodological framework. Int. J. Social Research Methodology.

[11] Page M.J. (2021). The PRISMA 2020 statement: an updated guideline for reporting systematic reviews. BMJ.

[12] Tricco A.C. (2018). PRISMA extension for scoping reviews (PRISMA-ScR): checklist and explanation. Ann. Intern. Med..

[13] Ouzzani M. (2016). Rayyan - a web and mobile app for systematic reviews. Syst. Rev..

[14] Braun V., Clarke V. (2012). APA Handbook of Research Methods in Psychology, Vol 2: Research Designs: Quantitative, Qualitative, Neuropsychological, and Biological.

[15] Richardson-Tench M., Martens E. (2005). From systems to tissues: a revolution in learning in perioperative education. Educ. Health.

[16] Kuiper R.A. (2004). Nursing reflections from journaling during a perioperative internship: the official voice of perioperative nursing. AORN J..

[17] Matapo R., Kennedy T. (2020). Back to the future an interprofessional 12-week OR orientation programme. Dissector.

[18] Fitzgerald B. (2009). Educating novice perioperative nurses. Perioperative Nursing Clinics.

[19] Byrd D. (2015). Implementing a perioperative RN training program for recent graduates. AORN J..

[20] DeKastle R.J. (2010). Onboarding: laying the foundation. OR Manag..

[21] Gorgone P.D. (2016). Development of a new graduate perioperative nursing program at an urban pediatric institution. AORN J..

[22] Martin K.K. (2011). Meeting the challenge of perioperative education: the official voice of perioperative nursing. AORN J..

[23] Osgood P., Hemingway M. (2019). Building a cardiac scrub simulation orientation program for new perioperative nurses. AORN J..

[24] Wilson G. (2012). Redesigning OR orientation. AORN J..

[25] Dajee M. (2002). Supporting newly qualified nurses in operating theatres. Nursing Times.

[26] Ray N. (2015). Graduate nurse to PACU nurse resident: developing an in-depth orientation for the graduate nurse. J. PeriAnesthesia Nurs..

[27] Graling P.R., Rusynko B. (2001). Implementing a perioperative nursing fellowship program: the official voice of perioperative nursing. AORN J..

[28] Saver C. (2014). Train, sustain, retain: targeted recruitment reduces hospital openings from 50 to 0. OR Manag..

[29] Latz P.A., Nordbye D. (2004). Educating nurses into the perioperative arena: the official voice of perioperative nursing. AORN J..

[30] Ceschini D. (2016). Innovative programs promoting careers in OR nursing. AORN J..

[31] Penprase B. (2000). Collaboratively developing an orientation program for OR nurses. AORN J..

[32] Saver C. (2014). Orientation redesign improves employee satisfaction, retention. OR Manag..

[33] Whelan T. (2016). Knowledge and skills enhacement through perioperative nursing simulation lab training. Ornac j.

[34] Punnara D., Barta M. (2009). Graduate nurses: learning how to feel the thrill. J. Vasc. Nurs..

[35] Wu G., Taylor B. (2020). Successful training programmes for nurses new to the perioperative environment. Dissector.

[36] Lin Y.E. (2020). Anxiety and work stress among newly employed nurses during the first year of a residency programme: a longitudinal study. J. Nurs. Manag..

[37] Eriksson J., Lindgren B.M., Lindahl E. (2020). Newly trained operating room nurses' experiences of nursing care in the operating room. Scand. J. Caring Sci..

[38] Nijkamp N., Foran P. (2021). The effects of staffing practices on safety and quality of perioperative nursing care - an integrative review. Journal of Perioperative Nursing.

[39] Hoffart N., Waddell A., Young M.B. (2011). A model of new nurse transition. J. Prof. Nurs..

[40] Akselbo I., Killingberg H., Aune I. (2020). Simulation as a pedagogical learning method for critical paediatric nursing in Bachelor of Nursing programmes: a qualitative study. Adv Simul (Lond).

[41] Ball K., Doyle D., Oocumma N.I. (2015). Nursing shortages in the OR: solutions for new models of education. AORN J..

[42] Beitz J.M. (2019). Addressing the perioperative nursing shortage through education: a perioperative imperative. AORN J..

[43] Dunn D. (2014). Where, oh where, are the OR nurses: orientation. OR Nurse.

[44] Bratt M. (2013). Nurse residency program: best practices for optimizing organizational success. Journal for Nurses in Professional Development.

